# Supplementary material for: Dynamic network coding of working-memory domains and working-memory processes
Source: Nat Commun. 2019 Feb 25;10:936. doi: 10.1038/s41467-019-08840-8 (PMC6389921; doi:10.1038/s41467-019-08840-8)
Supplement: Supplementary file 1 — Supplementary Information [file 41467_2019_8840_MOESM1_ESM.pdf]

DYNAMIC NETWORK CODING OF WORKING-MEMORY DOMAINS AND WORKING-MEMORY PROCESSES

Soreq et al.

1 BEHAVIORAL RESULTS

1.1 STUDY 1 ACCURACY

In Study 1, Accuracy (see Supplementary Table 1 and Supplementary Figure 1) was examined using a 3 x 3 (domain x load) repeated-measures ANOVA (see Supplementary Table 2). Mean accuracy was 91.13%±6.31*SDs*. There was a significant effect of load ($F_{2,36} = 11.85$, $p < 0.001$) with significantly lower accuracy during high compared to both low ($t = -4.859$, $p < 0.001$) and medium load ($t = -3.03$, $p < 0.018$). There was no significant main effect of domain ($F_{2,36} = 2.667$, $p < 0.085$) and no significant interaction between load and domain ($F_{4,72} = 1.98$, $p > 0.1$).

Load	Domain	meanACC	stdACC
Low	Number	0.9474	0.0971
Low	Fractal	0.9562	0.0755
Low	Position	0.9386	0.0996
Med	Number	0.9737	0.0625
Med	Fractal	0.8860	0.1476
Med	Position	0.9474	0.1929
High	Number	0.8158	0.2351
High	Fractal	0.7983	0.2122
High	Position	0.9386	0.1140

SUPPLEMENTARY TABLE 1

Study 1 summary statistics a 3 x 3 (domain x load) Design

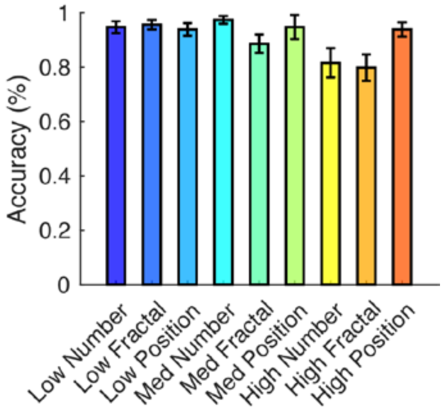

SUPPLEMENTARY FIGURE 1

Study 1 accuracy measures by experimental factors

Terms	SumSq	DF	MeanSq	F	pValueGG
(Intercept)	5112.4270	1	5112.4270	3959.7509	0.0001
Error	23.2398	18	1.2911	1.0000	0.5000
(Intercept):Load	11.3802	2	5.6901	11.8498	0.0010
Error(Load)	17.2866	36	0.4802	1.0000	0.5000
(Intercept):Domain	3.8714	2	1.9357	2.6670	0.0926
Error(Domain)	26.1287	36	0.7258	1.0000	0.5000
(Intercept):Load:Domain	7.0059	4	1.7515	1.9809	0.1346
Error(Load:Domain)	63.6609	72	0.8842	1.0000	0.5000

SUPPLEMENTARY TABLE 2

Accuracy was examined using a 3 x 3 (domain x load) repeated-measures ANOVA There was a significant main effect of load ($F_{2,36} = 11.85$, $p_{GG} < 0.0001$) There was no significant main effect of domain and no significant interaction between load and domain($F_{2,36} = 2.667$, $p = 0.083$; $F_{4,72} = 1.7515$, $p = 0.106$)

1.2 STUDY 1 REACTION TIME

Median reaction times (RT) (correct trials only) were 3.637*s* ± 1.54*SDs*. Reaction time (RT) effects were examined using repeated-measures ANOVA. Main effects for RT were found for both load and domain ($F_{2,36}$ = 168.59, $p < 0.0001$; $F_{2,36}$ = 24.5, $p < 0.0001$), as well as an interaction effect for domain by load ($F_{4,72}$ = 4.508, $p < 0.005$). Post-hoc tests revealed that RTs increased as a function of load ($t_{low<med}$ = −10.7, $p < 0.0001$; $t_{low<high}$ =−16.3, $p < 0.0001$). And both fractal and number trials were slower than location trial (location ¡ number ($t_{location<number}$ = 5.38, $p < 0.001$; $t_{location<fractal}$ = 5.88, $p < 0.001$). Together, these results demonstrate that the participants understood and could perform the task accurately and that they exhibited the expected costs of load on performance.

Terms	Load	Stimulus	meanLogRT	stdLogRT
Low Number	Low	Number	8.0749	0.1591
Low Fractal	Low	Fractal	8.0810	0.1905
Low Position	Low	Position	7.9074	0.2087
Med Number	Med	Number	8.3459	0.1555
Med Fractal	Med	Fractal	8.3780	0.1811
Med Position	Med	Position	8.0788	0.2242
High Number	High	Number	8.5894	0.1627
High Fractal	High	Fractal	8.5699	0.1639
High Position	High	Position	8.2701	0.2212

SUPPLEMENTARY TABLE 3

Study 2 summary statistics for RT with a 3 x 3 (domain x load) Design

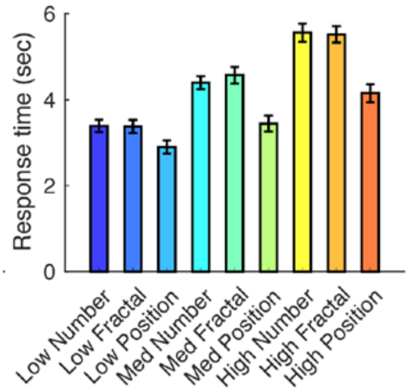

SUPPLEMENTARY FIGURE 2

Study 1 response time measures by experimental factors

Terms	SumSq	DF	MeanSq	F	pValue
(Intercept)	11652.7861	1	11652.7861	83286.7815	0.0001
Error	2.5185	18	0.1400	1.0000	0.5000
(Intercept):Load	5.9230	2	2.9615	176.8586	0.0001
Error(Load)	0.6029	36	0.0168	1.0000	0.5000
(Intercept):Stimulus	2.4611	2	1.2306	31.4765	0.0001
Error(Stimulus)	1.4074	36	0.0391	1.0000	0.5000
(Intercept):Load:Stimulus	0.1508	4	0.0377	2.3938	0.0584
Error(Load:Stimulus)	1.1335	72	0.0158	1.0000	0.5000

SUPPLEMENTARY TABLE 4

Reaction time was examined using a 3 x 3 (domain x load) repeated-measures ANOVA There was a significant main effect of load and domain ($F_{2,36}$ = 168.59, $p < 0.00001$; $F_{2,36}$ = 24.507, $p < 0.00001$) There was a significant interaction effect between load and domain ($F_{4,72}$ = 4.508, $p < 0.005$)

1.3 Study 2 Accuracy

In Study 2 accuracy (see Supplementary Table 5 and Supplementary Figure 3) was examined using a 2 x 2 x 2 (manipulation x domain x load) repeated- measures ANOVA (see Supplementary Table 6). Mean accuracy was 84.24% ± 8.5*SDs*. There was a significant main effect of load ($F_{1,15}$ = 43.2, p < 0.0001), and a significant interaction between domain and load ($F_{1,15}$ = 5.29, p < 0.04) with accuracy for number trials significantly lower during high-load.

Load	Stimulus	Manipulation	meanACC	stdACC
High	Pos	Off	0.8750	0.1291
High	Pos	On	0.8230	0.1287
High	Num	Off	0.9480	0.0798
High	Num	On	0.9063	0.0854
Low	Pos	Off	0.7917	0.0746
Low	Pos	On	0.7813	0.0798
Low	Num	Off	0.7188	0.1456
Low	Num	On	0.7605	0.1049

SUPPLEMENTARY TABLE 5

Study 2 summary statistics a 2 x 2 (domain x load x manipulation) design

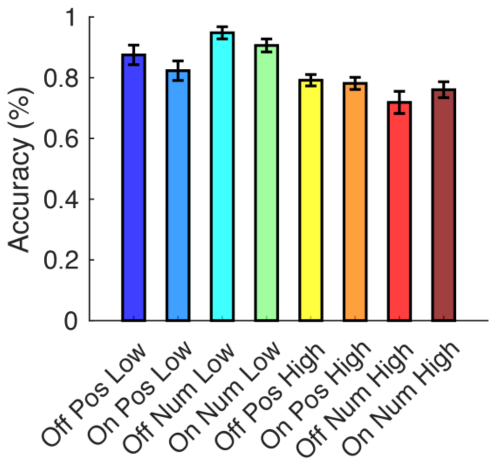

SUPPLEMENTARY FIGURE 3

Study 2 accuracy measures by experimental factors

Terms	SumSq	DF	MeanSq	F	pValueGG
(Intercept)	3140.2813	1	3140.2813	5252.0384	0.0001
Error	8.9688	15	0.5980	1.0000	0.5000
(Intercept):Load	18.0001	1	18.0001	43.2001	0.0001
Error(Load)	6.2500	15	0.4167	1.0000	0.5000
(Intercept):Stimulus	0.2813	1	0.2813	1.0630	0.3189
Error(Stimulus)	3.9688	15	0.2646	1.0000	0.5000
(Intercept):Manipulation	0.2813	1	0.2813	1.4211	0.2518
Error(Manipulation)	2.9688	15	0.1980	1.0000	0.5000
(Intercept):Load:Stimulus	4.5000	1	4.5000	5.2942	0.0362
Error(Load:Stimulus)	12.7500	15	0.8500	1.0000	0.5000
(Intercept):Load:Manipulation	1.1251	1	1.1251	2.7552	0.1178
Error(Load:Manipulation)	6.1250	15	0.4084	1.0000	0.5000
(Intercept):Stimulus:Manipulation	0.2813	1	0.2813	1.0630	0.3189
Error(Stimulus:Manipulation)	3.9688	15	0.2646	1.0000	0.5000
(Intercept):Load:Stimulus:Manipulation	0.1250	1	0.1250	0.4546	0.5105
Error(Load:Stimulus:Manipulation)	4.1250	15	0.2750	1.0000	0.5000

SUPPLEMENTARY TABLE 6

Accuracy was examined using a 2 x 2 X 2 (domain x load x manipulation) repeated-measures ANOVA There was a significant main effect of load ($F_{1,15}$ = 31.532, p < 0.00001) There was no significant main effect of domain or manipulation and no significant interactions.

1.4 Study 2 reaction time

Median reaction times (RT) (correct trials only) were 3.937s ± 1.26*SDs*. RTs were examined using a model with the same structure. There was a significant main effect of load and a significant interaction between load and domain ($F_{1,15} = 74.26$, $p < 0.0001$; $F_{1,15} = 17.05$, $p < 0.001$). RTs increased as a function of load ($t = 8.6$, $p < 0.0001$), and RTs for both number and location trials under low load were slower relative to high load ($t = 9.17$, $p < 0.001$; $t = 4.7$, $p < 0.001$). There were no significant effects of manipulation and no other significant interactions. Together, these results show that participants were engaged in the task, exhibited similar effects of load to Study 1, and that participants could successfully manipulate information in working memory.

Load	Stimulus	Manipulation	meanLogRT	stdLogRT
Low	Pos	Off	8.0674	0.2706
Low	Pos	On	8.1237	0.2912
Low	Num	Off	8.0254	0.2196
Low	Num	On	8.0030	0.2451
High	Pos	Off	8.3726	0.2694
High	Pos	On	8.2608	0.2546
High	Num	Off	8.4601	0.2888
High	Num	On	8.4559	0.2427

SUPPLEMENTARY TABLE 7

Study 2 summary statistics for RT with a 2 x 2 x 2 (domain x load x manipulation) design

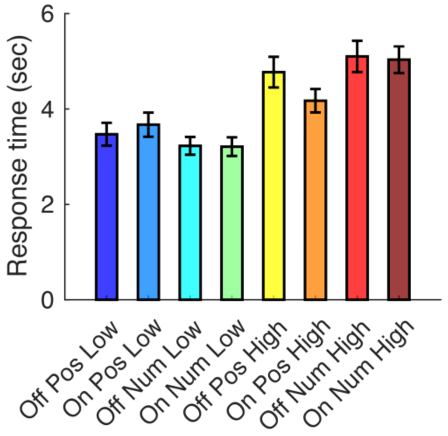

SUPPLEMENTARY FIGURE 4

Study 2 summary statistics for RT with a 2 x 2 x 2 (domain x load x manipulation) design

Terms	SumSq	DF	MeanSq	F	pValueGG
(Intercept)	8650.9647	1	8650.9647	22344.4373	0.0001
Error	5.8075	15	0.3872	1.0000	0.5000
(Intercept):Load	3.5372	1	3.5372	120.0365	0.0001
Error(Load)	0.4421	15	0.0295	1.0000	0.5000
(Intercept):Stimulus	0.0288	1	0.0288	0.8915	0.3601
Error(Stimulus)	0.4838	15	0.0323	1.0000	0.5000
(Intercept):Manipulation	0.0136	1	0.0136	0.7724	0.3934
Error(Manipulation)	0.2624	15	0.0175	1.0000	0.5000
(Intercept):Load:Stimulus	0.3966	1	0.3966	23.4223	0.0003
Error(Load:Stimulus)	0.2540	15	0.0170	1.0000	0.5000
(Intercept):Load:Manipulation	0.0450	1	0.0450	1.6124	0.2236
Error(Load:Manipulation)	0.4186	15	0.0280	1.0000	0.5000
(Intercept):Stimulus:Manipulation	0.0017	1	0.0017	0.1439	0.7098
Error(Stimulus:Manipulation)	0.1760	15	0.0118	1.0000	0.5000
(Intercept):Load:Stimulus:Manipulation	0.0694	1	0.0694	3.0314	0.1022
Error(Load:Stimulus:Manipulation)	0.3432	15	0.0229	1.0000	0.5000

SUPPLEMENTARY TABLE 8

Reaction time was examined using a 2 x 2 x 2 (domain x load x manipulation) repeated-measures ANOVA There were significant main effect of load and interaction between load and domain ($F_{1,15} = 74.262$, $p < 0.00001$; $F_{1,15} = 17.049$, $p < 0.001$) There were no other significant effects

2 PARCELLATION SETS

2.1 STAGE GENERAL ROI SET

ROIid	Volume	X	Y	Z	hemisphere	lobe	name WFU	name AAL2
1	1981	2	8	50	Right	Temporal	BA.32	Supp Motor Area L
2	1552	-30	-8	54	Left	Temporal	BA.06	Precentral L
3	1306	-46	4	30	Left	Temporal	BA.09	Precentral L
4	762	-20	-62	56	Left	Occipital	BA.07	Parietal Sup L
5	1171	30	-4	56	Right	Temporal	BA.06	Frontal Sup 2 R
6	1733	-36	-46	46	Left	Occipital	BA.40	Parietal Inf L
7	311	-12	-74	46	Left	Occipital	BA.07	Parietal Sup L
8	764	-22	-70	38	Left	Temporal	BA.07	Parietal Sup L
9	2650	26	-64	46	Right	Temporal	BA.07	Parietal Sup R
10	628	-24	-78	24	Left	Frontal	BA.19	Occipital Mid L
11	882	50	8	30	Right	Temporal	BA.09	Frontal Inf Oper R
12	799	40	-42	44	Right	Temporal	BA.40	Parietal Inf R
13	359	-32	18	2	Left	Cerebelum	BA.13	Insula L
14	76	-10	-4	72	Left	Frontal	BA.06	Supp Motor Area L
15	115	-48	24	26	Left	Insula	BA.46	Frontal Inf Tri L
16	98	52	-28	46	Right	Frontal	BA.02	Postcentral R
17	140	-10	-20	6	Left	Cerebelum	null	Thalamus L
18	166	48	28	22	Right	Temporal	BA.46	Frontal Inf Tri R
19	60	48	20	26	Right	Occipital	BA.46	Frontal Inf Tri R
20	138	0	-28	-4	Medial	Cerebelum	null	Thalamus R

SUPPLEMENTARY TABLE 9

Data-drive parcellation set based on Stage general conjunction.

2.2 Domain general ROI set

ROIid	Volume	X	Y	Z	hemisphere	lobe	name WFU	name AAL2
1	993	28	-76	30	Right	Occipital	BA.19	Occipital Sup R
2	1760	-20	-68	44	Left	Temporal	BA.07	Parietal Sup L
3	2090	30	-86	8	Right	Temporal	BA.19	Occipital Mid R
4	2163	-22	-90	8	Left	Temporal	BA.18	Occipital Mid L
5	2574	2	6	50	Right	Temporal	BA.06	Supp Motor Area L
6	518	-24	-78	24	Left	Frontal	BA.31	Occipital Mid L
7	2094	-30	-16	60	Left	Temporal	BA.06	Precentral L
8	1088	-46	4	32	Left	Temporal	BA.09	Precentral L
9	2375	28	-62	48	Right	Temporal	BA.07	Parietal Sup R
10	1429	48	14	28	Right	Temporal	BA.09	Frontal Inf Oper R
11	3872	-28	-70	-16	Left	Cerebelum	BA.19	Fusiform L
12	1879	32	-54	-20	Right	Cerebelum	BA.37	Fusiform R
13	2156	26	-76	-14	Right	Cerebelum	BA.18	Lingual R
14	656	-26	-6	52	Left	Temporal	BA.06	Precentral L
15	1126	32	-4	56	Right	Temporal	BA.06	Frontal Sup 2 R
16	1178	-32	-50	50	Left	Temporal	BA.40	Parietal Inf L
17	371	-50	-26	46	Left	Temporal	BA.02	Postcentral L
18	498	46	-62	-12	Right	Cerebelum	BA.37	Temporal Inf R
19	704	-6	-82	-6	Left	Cerebelum	BA.18	Lingual L
20	1218	8	-78	-2	Right	Cerebelum	BA.18	Lingual R
21	839	-40	-38	48	Left	Occipital	BA.40	Postcentral L
22	737	12	-92	6	Right	Temporal	BA.18	Calcarine R
23	469	-10	-22	2	Left	Cerebelum	BA.27	Thalamus L
24	389	-30	18	2	Left	Cerebelum	BA.13	Insula L
25	333	34	18	4	Right	Cerebelum	BA.13	Insula R
26	267	-8	-96	-2	Left	Cerebelum	BA.17	Calcarine L
27	221	12	-18	4	Right	Cerebelum	null	Thalamus R
28	257	6	-28	-6	Right	Cerebelum	null	Thalamus R
29	199	-4	-28	-8	Left	Cerebelum	null	Thalamus L
30	54	-48	-24	20	Left	Frontal	BA.13	Rolandic Oper L
31	112	46	-36	44	Right	Occipital	BA.40	SupraMarginal R
32	93	-44	30	24	Left	Occipital	BA.46	Frontal Inf Tri L
33	84	-16	4	2	Left	Cerebelum	null	Putamen L

SUPPLEMENTARY TABLE 10

Data-drive parcellation set based on Domain general conjunction.

2.3 WHOLE BRAIN ROI SET

ROIid	Volume	X	Y	Z	hemisphere	lobe	name Yeo7	name AAL2
1	625	16	56	-18	Right	Frontal	Limbic	OFCant R
2	426	10	16	-20	Right	Frontal	Limbic	Rectus R
3	521	6	34	-18	Right	Frontal	Limbic	Rectus R
4	301	16	34	-24	Right	Frontal	Limbic	OFCmed R
5	783	10	46	-2	Right	Frontal	Default	Frontal Med Orb R
6	712	18	64	2	Right	Frontal	Default	Frontal Sup 2 R
7	607	32	54	-4	Right	Frontal	Frontoparietal	Frontal Mid 2 R
8	652	46	46	-6	Right	Frontal	Frontoparietal	Frontal Mid 2 R
9	1001	30	50	18	Right	Frontal	Frontoparietal	Frontal Sup 2 R
10	925	10	52	22	Right	Frontal	Default	Frontal Sup Medial R
11	792	38	34	30	Right	Frontal	Frontoparietal	Frontal Mid 2 R
12	1050	16	36	48	Right	Frontal	Default	Frontal Sup 2 R
13	683	26	30	36	Right	Frontal	Default	Frontal Sup 2 R
14	747	42	14	48	Right	Frontal	Frontoparietal	Frontal Mid 2 R
15	622	8	20	30	Right	Limbic	Ventral Attention	Cingulate Mid R
16	947	54	24	0	Right	Frontal	Default	Frontal Inf Tri R
17	708	34	36	-18	Right	Frontal	Frontoparietal	OFCant R
18	414	28	18	-22	Right	Frontal	Limbic	OFCpost R
19	795	50	34	14	Right	Frontal	Frontoparietal	Frontal Inf Tri R
20	473	38	20	6	Right	Insula	Ventral Attention	Insula R
21	888	56	8	22	Right	Frontal	Dorsal Attention	Frontal Inf Oper R
22	580	40	16	28	Right	Frontal	Frontoparietal	Frontal Inf Oper R
23	930	58	-10	26	Right	Parietal	Somatomotor	Postcentral R
24	535	8	-24	64	Right	Frontal	Somatomotor	Supp Motor Area R
25	444	8	-10	52	Right	Frontal	Somatomotor	Supp Motor Area R
26	727	28	-14	66	Right	Frontal	Somatomotor	Precentral R
27	603	50	-6	46	Right	Frontal	Somatomotor	Precentral R
28	474	8	14	48	Right	Frontal	Ventral Attention	Supp Motor Area R
29	903	14	6	64	Right	Frontal	Ventral Attention	Supp Motor Area R
30	597	26	12	48	Right	Frontal	Frontoparietal	Frontal Sup 2 R
31	432	40	2	34	Right	Frontal	Dorsal Attention	Precentral R
32	410	34	-6	50	Right	Frontal	Dorsal Attention	Precentral R
33	869	42	-24	52	Right	Parietal	Somatomotor	Postcentral R
34	463	42	4	-8	Right	Insula	Ventral Attention	Insula R
35	460	42	2	6	Right	Insula	Ventral Attention	Insula R
36	519	38	20	-12	Right	Insula	Frontoparietal	Insula R
37	565	40	-14	-2	Right	Insula	Somatomotor	Insula R
38	607	34	-40	48	Right	Parietal	Dorsal Attention	Postcentral R
39	838	22	-34	68	Right	Parietal	Somatomotor	Postcentral R
40	480	44	-12	12	Right	Frontal	Somatomotor	Rolandic Oper R

ROIid	Volume	X	Y	Z	hemisphere	lobe	name Yeo7	name AAL2
41	836	26	-56	66	Right	Parietal	Dorsal Attention	Parietal Sup R
42	562	16	-70	34	Right	Parietal	Frontoparietal	Precuneus R
43	1457	32	-62	48	Right	Parietal	Dorsal Attention	Parietal Sup R
44	1001	8	-58	58	Right	Parietal	Dorsal Attention	Precuneus R
45	1222	54	-28	40	Right	Parietal	Dorsal Attention	SupraMarginal R
46	949	58	-30	18	Right	Temporal	Ventral Attention	Temporal Sup R
47	1180	56	-46	36	Right	Parietal	Frontoparietal	SupraMarginal R
48	965	48	-62	34	Right	Parietal	Default	Angular R
49	780	42	-76	26	Right	Occipital	Dorsal Attention	Occipital Mid R
50	752	50	-60	14	Right	Temporal	Dorsal Attention	Temporal Mid R
51	389	28	10	-42	Right	Temporal	Limbic	Temporal Pole Mid R
52	507	42	18	-36	Right	Temporal	Limbic	Temporal Pole Mid R
53	857	54	10	-22	Right	Temporal	Default	Temporal Pole Sup R
54	339	52	-34	-2	Right	Temporal	Default	Temporal Mid R
55	690	62	-24	-24	Right	Temporal	Default	Temporal Inf R
56	488	56	-8	-32	Right	Temporal	Default	Temporal Inf R
57	467	48	2	-40	Right	Temporal	Limbic	Temporal Inf R
58	421	42	-12	-36	Right	Occipital	Limbic	Fusiform R
59	392	44	-28	-26	Right	Occipital	Limbic	Fusiform R
60	388	32	0	-46	Right	Occipital	Limbic	Fusiform R
61	1033	60	-4	2	Right	Temporal	Somatomotor	Temporal Sup R
62	511	40	-26	14	Right	Frontal	Somatomotor	Rolandic Oper R
63	816	62	-24	-4	Right	Temporal	Somatomotor	Temporal Sup R
64	757	58	-10	-16	Right	Temporal	Default	Temporal Mid R
65	961	60	-44	8	Right	Temporal	Ventral Attention	Temporal Mid R
66	444	48	-60	-16	Right	Temporal	Visual	Temporal Inf R
67	577	38	-70	-18	Right	Occipital	Visual	Fusiform R
68	622	26	-46	-14	Right	Occipital	Visual	Fusiform R
69	688	56	-58	-6	Right	Temporal	Dorsal Attention	Temporal Mid R
70	974	62	-44	-18	Right	Temporal	Frontoparietal	Temporal Inf R
71	610	42	-46	-24	Right	Occipital	Visual	Fusiform R
72	635	22	-64	-10	Right	Occipital	Visual	Lingual R
73	624	32	-84	20	Right	Occipital	Visual	Occipital Mid R
74	829	46	-76	2	Right	Occipital	Visual	Occipital Mid R
75	874	20	-82	38	Right	Occipital	Visual	Occipital Sup R
76	761	20	-84	-12	Right	Occipital	Visual	Lingual R
77	521	12	-76	24	Right	Occipital	Visual	Cuneus R
78	691	24	-96	6	Right	Occipital	Visual	Occipital Mid R
79	462	8	-76	-4	Right	Occipital	Visual	Lingual R
80	696	10	-90	10	Right	Occipital	Visual	Calcarine R
81	649	32	-92	-12	Right	Occipital	Visual	Occipital Inf R

ROIid	Volume	X	Y	Z	hemisphere	lobe	name Yeo7	name AAL2
82	650	16	-70	8	Right	Occipital	Visual	Calcarine R
83	576	10	34	16	Right	Limbic	Default	Cingulate Ant R
84	362	6	-2	34	Right	Limbic	Ventral Attention	Cingulate Mid R
85	394	6	-40	26	Right	Limbic	Default	Cingulate Post R
86	612	14	-58	18	Right	Parietal	Default	Precuneus R
87	273	30	-54	6	Right	Occipital	Visual	Calcarine R
88	259	8	-20	30	Right	Limbic	Frontoparietal	Cingulate Mid R
89	420	10	-24	44	Right	Limbic	Somatomotor	Cingulate Mid R
90	685	8	-58	38	Right	Parietal	Default	Precuneus R
91	545	10	-40	48	Right	Parietal	Ventral Attention	Precuneus R
92	444	32	2	-22	Right	Limbic	Limbic	Amygdala R
93	334	30	-38	-2	Right	Limbic	Visual	Hippocampus R
94	295	36	-16	-20	Right	Limbic	Limbic	Hippocampus R
95	558	30	-30	-14	Right	Limbic	Visual	ParaHippocampal R
96	364	30	-20	-28	Right	Limbic	Limbic	ParaHippocampal R
97	377	26	-4	-32	Right	Limbic	Limbic	ParaHippocampal R
98	631	16	-48	2	Right	Occipital	Visual	Lingual R
99	621	20	-8	-16	Right	Limbic	Limbic	Hippocampus R
100	684	34	-80	-42	Right	Cerebelum	Default	Cerebelum Crus2 R
101	621	8	-52	-14	Right	Cerebelum	Somatomotor	Vermis 4 5
102	962	40	-76	-30	Right	Cerebelum	Default	Cerebelum Crus1 R
103	442	14	-42	-26	Right	Cerebelum	Limbic	Cerebelum 4 5 R
104	207	24	-36	-44	Right	Cerebelum	Limbic	Cerebelum 10 R
105	352	8	-70	-38	Right	Cerebelum	Ventral Attention	Cerebelum 8 R
106	483	8	-70	-22	Right	Cerebelum	Ventral Attention	Cerebelum 6 R
107	635	46	-48	-44	Right	Cerebelum	Default	Cerebelum Crus2 R
108	408	18	-48	-54	Right	Cerebelum	Somatomotor	Cerebelum 9 R
109	537	24	-60	-54	Right	Cerebelum	Ventral Attention	Cerebelum 8 R
110	527	22	-56	-24	Right	Cerebelum	Somatomotor	Cerebelum 6 R
111	680	12	-86	-36	Right	Cerebelum	Default	Cerebelum Crus2 R
112	613	20	-74	-52	Right	Cerebelum	Dorsal Attention	Cerebelum 8 R
113	568	38	-58	-34	Right	Cerebelum	Frontoparietal	Cerebelum Crus1 R
114	583	24	-72	-30	Right	Cerebelum	Default	Cerebelum Crus1 R
115	393	8	-58	-52	Right	Cerebelum	Default	Cerebelum 9 R
116	934	44	-66	-50	Right	Cerebelum	Frontoparietal	Cerebelum Crus2 R
117	330	8	-54	-36	Right	Cerebelum	Default	Cerebelum 9 R
118	481	34	-48	-56	Right	Cerebelum	Ventral Attention	Cerebelum 8 R
119	473	32	-38	-32	Right	Cerebelum	Ventral Attention	Cerebelum 6 R
120	200	24	-38	24	Right	subcorticalGM	null	Caudate R
121	474	14	12	10	Right	subcorticalGM	Frontoparietal	Caudate R
122	319	16	-6	20	Right	subcorticalGM	Frontoparietal	Caudate R

ROIid	Volume	X	Y	Z	hemisphere	lobe	name Yeo7	name AAL2
123	453	14	20	-2	Right	subcorticalGM	Default	Caudate R
124	496	28	6	0	Right	subcorticalGM	Ventral Attention	Putamen R
125	624	16	8	-10	Right	subcorticalGM	Limbic	Putamen R
126	465	12	-28	-2	Right	subcorticalGM	Visual	Thalamus R
127	391	14	-28	12	Right	subcorticalGM	null	Thalamus R
128	526	6	-10	4	Right	subcorticalGM	null	Thalamus R
129	278	6	-38	-54	Right	null	Ventral Attention	null
130	333	10	-20	-32	Right	null	Limbic	null
131	187	8	-24	-46	Right	null	Limbic	null
132	431	8	-26	-18	Right	Cerebelum	Limbic	Cerebelum 3 R
133	316	8	-36	-38	Right	Cerebelum	Limbic	Cerebelum 9 R
134	391	-4	28	-10	Left	Limbic	Limbic	Cingulate Ant L
135	363	-18	18	-22	Left	Frontal	Limbic	OFCmed L
136	263	-4	18	-22	Left	Frontal	Limbic	Rectus L
137	588	-6	38	-22	Left	Frontal	Limbic	Rectus L
138	583	-6	48	-6	Left	Frontal	Default	Frontal Med Orb L
139	682	-18	56	-16	Left	Frontal	Limbic	OFCant L
140	721	-4	48	10	Left	Frontal	Default	Frontal Sup Medial L
141	711	-10	64	2	Left	Frontal	Default	Frontal Sup 2 L
142	511	-28	54	2	Left	Frontal	Frontoparietal	Frontal Sup 2 L
143	574	-42	46	-8	Left	Frontal	Default	Frontal Mid 2 L
144	779	-28	50	20	Left	Frontal	Frontoparietal	Frontal Mid 2 L
145	702	-10	54	30	Left	Frontal	Default	Frontal Sup Medial L
146	976	-26	34	36	Left	Frontal	Ventral Attention	Frontal Mid 2 L
147	880	-46	28	26	Left	Frontal	Frontoparietal	Frontal Inf Tri L
148	1059	-10	34	50	Left	Frontal	Default	Frontal Sup 2 L
149	855	-38	16	46	Left	Frontal	Default	Frontal Mid 2 L
150	572	-4	16	46	Left	Frontal	Ventral Attention	Supp Motor Area L
151	776	-46	28	-10	Left	Frontal	Default	Frontal Inf Orb 2 L
152	499	-28	34	-16	Left	Frontal	Frontoparietal	OFCant L
153	485	-32	20	-16	Left	Frontal	Default	OFCpost L
154	769	-42	40	10	Left	Frontal	Frontoparietal	Frontal Inf Tri L
155	562	-32	22	4	Left	Insula	Ventral Attention	Insula L
156	837	-52	18	10	Left	Frontal	Default	Frontal Inf Tri L
157	840	-46	6	28	Left	Frontal	Frontoparietal	Precentral L
158	621	-40	-16	44	Left	Parietal	Somatomotor	Postcentral L
159	646	-58	-6	26	Left	Parietal	Somatomotor	Postcentral L
160	222	-16	-20	68	Left	Parietal	Somatomotor	Paracentral Lobule L
161	546	-6	-6	46	Left	Limbic	Somatomotor	Cingulate Mid L
162	905	-8	0	64	Left	Frontal	Ventral Attention	Supp Motor Area L
163	882	-56	-4	6	Left	Temporal	Somatomotor	Temporal Sup L

ROIid	Volume	X	Y	Z	hemisphere	lobe	name Yeo7	name AAL2
164	925	-22	10	52	Left	Frontal	Frontoparietal	Frontal Sup 2 L
165	681	-44	-2	48	Left	Frontal	Dorsal Attention	Precentral L
166	443	-26	-10	54	Left	Frontal	Dorsal Attention	Precentral L
167	895	-34	-22	64	Left	Parietal	Somatomotor	Postcentral L
168	511	-38	0	8	Left	Insula	Ventral Attention	Insula L
169	505	-38	8	-6	Left	Insula	Ventral Attention	Insula L
170	571	-36	-14	-2	Left	Insula	Ventral Attention	Insula L
171	841	-50	-24	40	Left	Parietal	Dorsal Attention	Postcentral L
172	422	-22	-32	62	Left	Parietal	Somatomotor	Postcentral L
173	440	-40	-16	12	Left	Frontal	Somatomotor	Rolandic Oper L
174	826	-6	-34	66	Left	Parietal	Somatomotor	Paracentral Lobule L
175	1219	-22	-56	64	Left	Parietal	Dorsal Attention	Parietal Sup L
176	734	-8	-72	30	Left	Occipital	Visual	Cuneus L
177	976	-28	-64	40	Left	Parietal	Dorsal Attention	Parietal Inf L
178	1060	-8	-68	54	Left	Parietal	Dorsal Attention	Precuneus L
179	767	-34	-40	46	Left	Parietal	Dorsal Attention	Parietal Inf L
180	548	-42	-32	14	Left	Temporal	Somatomotor	Temporal Sup L
181	855	-58	-26	18	Left	Parietal	Ventral Attention	SupraMarginal L
182	947	-42	-66	40	Left	Parietal	Default	Angular L
183	942	-50	-58	20	Left	Temporal	Default	Temporal Mid L
184	1259	-52	-44	38	Left	Parietal	Frontoparietal	Parietal Inf L
185	349	-36	6	-38	Left	Temporal	Limbic	Temporal Inf L
186	475	-34	18	-34	Left	Temporal	Limbic	Temporal Pole Sup L
187	363	-48	10	-32	Left	Temporal	Default	Temporal Pole Mid L
188	749	-48	6	-16	Left	Temporal	Default	Temporal Pole Sup L
189	325	-22	8	-40	Left	Temporal	Limbic	Temporal Pole Mid L
190	536	-56	-8	-24	Left	Temporal	Default	Temporal Mid L
191	859	-58	-30	2	Left	Temporal	Default	Temporal Mid L
192	943	-56	-48	4	Left	Temporal	Default	Temporal Mid L
193	756	-58	-28	-20	Left	Temporal	Default	Temporal Mid L
194	373	-48	-6	-38	Left	Temporal	Limbic	Temporal Inf L
195	299	-36	-14	-30	Left	Temporal	Limbic	Temporal Inf L
196	313	-50	-20	-30	Left	Temporal	Limbic	Temporal Inf L
197	757	-56	-16	-10	Left	Temporal	Default	Temporal Mid L
198	493	-26	-44	-18	Left	Occipital	Visual	Fusiform L
199	1064	-60	-50	-16	Left	Temporal	Frontoparietal	Temporal Inf L
200	442	-42	-54	-18	Left	Occipital	Dorsal Attention	Fusiform L
201	517	-46	-40	-26	Left	Temporal	Dorsal Attention	Temporal Inf L
202	349	-28	-6	-42	Left	Occipital	Limbic	Fusiform L
203	906	-40	-76	22	Left	Occipital	Visual	Occipital Mid L
204	593	-30	-88	12	Left	Occipital	Visual	Occipital Mid L

ROIid	Volume	X	Y	Z	hemisphere	lobe	name Yeo7	name AAL2
205	599	-16	-52	0	Left	Occipital	Visual	Lingual L
206	578	-42	-72	-14	Left	Occipital	Visual	Occipital Inf L
207	744	-24	-64	-14	Left	Occipital	Visual	Fusiform L
208	1196	-16	-86	32	Left	Occipital	Visual	Occipital Sup L
209	771	-48	-68	0	Left	Temporal	Dorsal Attention	Temporal Mid L
210	527	-34	-86	-4	Left	Occipital	Visual	Occipital Mid L
211	571	-8	-72	-2	Left	Occipital	Visual	Lingual L
212	895	-10	-100	6	Left	Occipital	Visual	Calcarine L
213	975	-14	-84	-14	Left	Occipital	Visual	Lingual L
214	649	-22	-98	-12	Left	Occipital	Visual	Occipital Inf L
215	753	-4	-82	12	Left	Occipital	Visual	Calcarine L
216	461	-22	-68	6	Left	Occipital	Visual	Calcarine L
217	296	-20	-42	18	Left	Parietal	Ventral Attention	Precuneus L
218	530	-6	-24	46	Left	Limbic	Somatomotor	Cingulate Mid L
219	729	-4	34	26	Left	Limbic	Default	Cingulate Ant L
220	183	-2	-6	32	Left	Limbic	Frontoparietal	Cingulate Mid L
221	482	-4	12	28	Left	Limbic	Ventral Attention	Cingulate Mid L
222	699	-8	-60	16	Left	Parietal	Default	Precuneus L
223	365	-4	-38	32	Left	Limbic	Default	Cingulate Mid L
224	291	-6	-20	26	Left	Limbic	Default	Cingulate Mid L
225	650	-6	-56	36	Left	Parietal	Default	Precuneus L
226	608	-8	-44	50	Left	Parietal	Ventral Attention	Precuneus L
227	223	-6	-44	12	Left	Limbic	Default	Cingulate Post L
228	519	-26	2	-20	Left	Limbic	Limbic	Amygdala L
229	295	-20	-38	4	Left	Limbic	null	Hippocampus L
230	314	-32	-42	-4	Left	Limbic	Visual	Hippocampus L
231	602	-22	-14	-18	Left	Limbic	Limbic	Hippocampus L
232	318	-34	-26	-16	Left	Limbic	Limbic	Hippocampus L
233	447	-20	-32	-12	Left	Limbic	Default	ParaHippocampal L
234	400	-30	-24	-28	Left	Occipital	Limbic	Fusiform L
235	405	-20	-6	-30	Left	Limbic	Limbic	ParaHippocampal L
236	407	-6	-68	-38	Left	Cerebelum	Ventral Attention	Cerebelum 8 L
237	347	-8	-52	-40	Left	Cerebelum	Default	Cerebelum 9 L
238	555	-36	-54	-32	Left	Cerebelum	Ventral Attention	Cerebelum Crus1 L
239	324	-8	-56	-54	Left	Cerebelum	Default	Cerebelum 9 L
240	488	-20	-72	-50	Left	Cerebelum	Dorsal Attention	Cerebelum 8 L
241	1025	-38	-76	-30	Left	Cerebelum	Default	Cerebelum Crus1 L
242	663	-30	-82	-42	Left	Cerebelum	Default	Cerebelum Crus2 L
243	325	-18	-48	-54	Left	Cerebelum	Somatomotor	Cerebelum 9 L
244	677	-6	-52	-12	Left	Cerebelum	Somatomotor	Cerebelum 4 5 L
245	387	-26	-36	-32	Left	Cerebelum	Ventral Attention	Cerebelum 4 5 L

ROIid	Volume	X	Y	Z	hemisphere	lobe	name Yeo7	name AAL2
246	844	-42	-64	-48	Left	Cerebelum	Frontoparietal	Cerebelum Crus2 L
247	716	-10	-82	-34	Left	Cerebelum	Frontoparietal	Cerebelum Crus2 L
248	590	-6	-70	-20	Left	Cerebelum	Ventral Attention	Cerebelum 6 L
249	484	-34	-52	-56	Left	Cerebelum	Ventral Attention	Cerebelum 8 L
250	398	-22	-60	-50	Left	Cerebelum	Ventral Attention	Cerebelum 8 L
251	457	-10	-40	-26	Left	Cerebelum	Limbic	Cerebelum 4 5 L
252	572	-46	-48	-44	Left	Cerebelum	Frontoparietal	Cerebelum Crus1 L
253	650	-26	-70	-32	Left	Cerebelum	Frontoparietal	Cerebelum Crus1 L
254	425	-20	-54	-24	Left	Cerebelum	Somatomotor	Cerebelum 6 L
255	332	-6	-56	-28	Left	Cerebelum	Limbic	Cerebelum 4 5 L
256	223	-24	-38	-46	Left	Cerebelum	Ventral Attention	Cerebelum 10 L
257	194	-8	24	10	Left	subcorticalGM	null	Caudate L
258	402	-12	10	8	Left	subcorticalGM	Default	Caudate L
259	847	-10	10	-10	Left	subcorticalGM	Limbic	Caudate L
260	298	-14	-4	20	Left	subcorticalGM	Frontoparietal	Caudate L
261	493	-24	4	-2	Left	subcorticalGM	Frontoparietal	Putamen L
262	430	-8	-26	-2	Left	subcorticalGM	null	Thalamus L
263	590	-4	-12	4	Left	subcorticalGM	null	Thalamus L
264	385	-10	-26	14	Left	subcorticalGM	Frontoparietal	Thalamus L
265	475	-4	-22	-16	Left	Cerebelum	null	Cerebelum 3 L
266	352	-4	-38	-54	Left	Cerebelum	Default	Cerebelum 9 L
267	352	-6	-34	-40	Left	Cerebelum	Limbic	Cerebelum 10 L
268	331	-4	-20	-38	Left	null	Limbic	null

SUPPLEMENTARY TABLE 11

External parcellation set (Shen & Finn 2015).

3 GLOBAL EFFECTS

3.1 WORKING-MEMORY MAINTENANCE EVOKES A LOW-ACTIVITY HIGH-CONNECTIVITY STATE

To estimate dynamic functional connectivity (FC), single subject models were constructed using gPPI, in which the task-related changes in cross correlations between each pair of ROIs was estimated for each stage of each individual trial while accounting for confounds (i.e., movements, the mean cross correlation between each pair, and task-related change in activation magnitude see above). The resultant PPI parameter estimates were taken to the group level, creating stacks of observations with identical dimensions to those defined for activation, but with each observation having a greater number of features.

Both activity and connectivity estimates were averaged across load, domain and runs producing a stack of subject x stage observations. Activity estimates were averaged across each HCL cluster. Connectivity estimates were averaged for all pairs of ROIs **within** each HCL cluster, producing five intra-cluster summary measures of dynamic connectivity for each of the three WM stages. This process was then repeated for FC estimates for ROIs pairs **between** different HCL clusters, producing ten summary estimates of inter-cluster dynamic connectivity for each WM state.

Study	Cluster	Mean	Std	t	df	Sig	P FDR	ci	
Study1	FPT	0.2711	0.0899	13.1459	18	0.0000	0.0000	0.2278	0.3144
Study2	FPT	0.0615	0.1015	2.4239	15	0.0285	0.0356	0.0074	0.1156
Study1	SMT	-0.1792	0.1570	-4.9764	18	0.0001	0.0002	-0.2549	-0.1035
Study2	SMT	-0.1344	0.1425	-3.7723	15	0.0018	0.0026	-0.2103	-0.0585
Study1	IPT	0.0540	0.1303	1.8075	18	0.0874	0.0874	-0.0088	0.1168
Study2	IPT	-0.0598	0.1148	-2.0813	15	0.0549	0.0611	-0.1210	0.0014
Study1	AVS	0.9721	0.2579	16.4272	18	0.0000	0.0000	0.8478	1.0964
Study2	AVS	0.5709	0.1138	20.0722	15	0.0000	0.0000	0.5103	0.6316
Study1	PVS	1.3095	0.4537	12.5817	18	0.0000	0.0000	1.0908	1.5281
Study2	PVS	0.7293	0.2891	10.0916	15	0.0000	0.0000	0.5753	0.8834

SUPPLEMENTARY TABLE 12

Paired measure t-test was performed on the difference between activation in Encode and Maintain over the FPT, SMT, IPT, AVS and PVS clusters.

Study	Stage	Cluster	Mean	Std	t	df	Sig	P FDR	ci	
Study1	Encode	FPT	0.6080	0.1735	15.2742	18	0.0000	0.0000	0.5244	0.6916
Study2	Encode	FPT	0.5223	0.1763	11.8530	15	0.0000	0.0000	0.4284	0.6163
Study1	Maintain	FPT	0.3369	0.1367	10.7448	18	0.0000	0.0000	0.2710	0.4028
Study2	Maintain	FPT	0.4608	0.1432	12.8760	15	0.0000	0.0000	0.3845	0.5371
Study1	Encode	SMT	0.0435	0.2683	0.7060	18	0.4892	0.5149	-0.0859	0.1728
Study2	Encode	SMT	0.0460	0.1986	0.9260	15	0.3691	0.4101	-0.0599	0.1518
Study1	Maintain	SMT	0.2227	0.2023	4.7974	18	0.0001	0.0002	0.1252	0.3202
Study2	Maintain	SMT	0.1804	0.1311	5.5052	15	0.0001	0.0001	0.1105	0.2502
Study1	Encode	IPT	0.2299	0.1557	6.4381	18	0.0000	0.0000	0.1549	0.3050
Study2	Encode	IPT	0.1496	0.1774	3.3745	15	0.0042	0.0060	0.0551	0.2441
Study1	Maintain	IPT	0.1759	0.1237	6.1977	18	0.0000	0.0000	0.1163	0.2356
Study2	Maintain	IPT	0.2094	0.1076	7.7821	15	0.0000	0.0000	0.1520	0.2667
Study1	Encode	AVS	0.9143	0.2303	17.3020	18	0.0000	0.0000	0.8033	1.0253
Study2	Encode	AVS	0.5812	0.1541	15.0893	15	0.0000	0.0000	0.4991	0.6633
Study1	Maintain	AVS	-0.0578	0.0906	-2.7781	18	0.0124	0.0165	-0.1015	-0.0141
Study2	Maintain	AVS	0.0102	0.0996	0.4108	15	0.6871	0.6871	-0.0428	0.0633
Study1	Encode	PVS	1.2437	0.4717	11.4926	18	0.0000	0.0000	1.0163	1.4711
Study2	Encode	PVS	0.6785	0.2461	11.0264	15	0.0000	0.0000	0.5473	0.8096
Study1	Maintain	PVS	-0.0658	0.1919	-1.4937	18	0.1526	0.1907	-0.1582	0.0267
Study2	Maintain	PVS	-0.0509	0.1876	-1.0846	15	0.2952	0.3473	-0.1508	0.0491

SUPPLEMENTARY TABLE 13

1-sample t-test was performed on the mean activity of both Encode and Maintain over the FPT, SMT, IPT, AVS and PVS clusters.

Study	Cluster	Mean	Std	t	df	Sig	ci	
Study1	FPT FPT	0.0025	0.0200	0.5475	18	0.5908	-0.0071	0.0122
Study1	SMT SMT	-0.0359	0.0322	-4.8606	18	0.0001	-0.0514	-0.0204
Study1	IPT IPT	-0.0023	0.0148	-0.6795	18	0.5054	-0.0094	0.0048
Study1	AVS AVS	0.0249	0.0212	5.1281	18	0.0001	0.0147	0.0351
Study1	PVS PVS	0.0177	0.0294	2.6273	18	0.0171	0.0035	0.0319
Study2	FPT FPT	0.0010	0.0295	0.1321	15	0.8967	-0.0148	0.0167
Study2	SMT SMT	-0.0294	0.0254	-4.6427	15	0.0003	-0.0430	-0.0159
Study2	IPT IPT	-0.0088	0.0124	-2.8422	15	0.0124	-0.0154	-0.0022
Study2	AVS AVS	0.0057	0.0322	0.7064	15	0.4908	-0.0115	0.0228
Study2	PVS PVS	0.0081	0.0399	0.8110	15	0.4300	-0.0132	0.0294

SUPPLEMENTARY TABLE 14

Paired measure t-test was performed on the difference between Encode and Maintain over the intra-FC clusters.

Study	Stage	Cluster	Mean	Std	t	df	Sig	ci	
Study1	Encode	FPT FPT	0.0795	0.0175	19.8167	18	0.0000	0.0710	0.0879
Study1	Maintain	FPT FPT	0.0770	0.0145	23.0916	18	0.0000	0.0700	0.0840
Study1	Encode	SMT SMT	0.0475	0.0306	6.7564	18	0.0000	0.0327	0.0622
Study1	Maintain	SMT SMT	0.0834	0.0209	17.4031	18	0.0000	0.0733	0.0934
Study1	Encode	IPT IPT	0.0538	0.0138	17.0619	18	0.0000	0.0472	0.0605
Study1	Maintain	IPT IPT	0.0561	0.0109	22.5119	18	0.0000	0.0509	0.0614
Study1	Encode	AVS AVS	0.1286	0.0217	25.7909	18	0.0000	0.1182	0.1391
Study1	Maintain	AVS AVS	0.1037	0.0157	28.7823	18	0.0000	0.0961	0.1113
Study1	Encode	PVS PVS	0.1352	0.0232	25.4523	18	0.0000	0.1241	0.1464
Study1	Maintain	PVS PVS	0.1175	0.0169	30.2562	18	0.0000	0.1094	0.1257
Study2	Encode	FPT FPT	0.0830	0.0230	14.4377	15	0.0000	0.0708	0.0953
Study2	Maintain	FPT FPT	0.0821	0.0199	16.4948	15	0.0000	0.0715	0.0927
Study2	Encode	SMT SMT	0.0441	0.0280	6.3083	15	0.0000	0.0292	0.0590
Study2	Maintain	SMT SMT	0.0736	0.0235	12.5058	15	0.0000	0.0610	0.0861
Study2	Encode	IPT IPT	0.0504	0.0080	25.0667	15	0.0000	0.0461	0.0547
Study2	Maintain	IPT IPT	0.0593	0.0107	22.1107	15	0.0000	0.0535	0.0650
Study2	Encode	AVS AVS	0.1095	0.0253	17.2996	15	0.0000	0.0960	0.1230
Study2	Maintain	AVS AVS	0.1038	0.0154	27.0395	15	0.0000	0.0956	0.1120
Study2	Encode	PVS PVS	0.1320	0.0271	19.4688	15	0.0000	0.1176	0.1465
Study2	Maintain	PVS PVS	0.1239	0.0246	20.1666	15	0.0000	0.1108	0.1370

SUPPLEMENTARY TABLE 15

1-sample t-test was perfomed on the mean intra-connectivity of both Encode and Maintain over the intra-FC clusters.

Study	Cluster	Mean	Std	t	df	Sig	ci	
Study1	FPT SMT	-0.0360	0.0172	-9.1110	18	0.0000	-0.0443	-0.0277
Study1	FPT IPT	-0.0043	0.0136	-1.3809	18	0.1842	-0.0109	0.0022
Study1	FPT AVS	0.0243	0.0207	5.1051	18	0.0001	0.0143	0.0343
Study1	FPT PVS	0.0312	0.0218	6.2399	18	0.0000	0.0207	0.0417
Study1	SMT IPT	-0.0234	0.0134	-7.6132	18	0.0000	-0.0299	-0.0170
Study1	SMT AVS	-0.0363	0.0204	-7.7533	18	0.0000	-0.0461	-0.0264
Study1	SMT PVS	-0.0266	0.0243	-4.7609	18	0.0002	-0.0383	-0.0149
Study1	IPT AVS	0.0064	0.0172	1.6294	18	0.1206	-0.0019	0.0147
Study1	IPT PVS	0.0068	0.0179	1.6493	18	0.1164	-0.0019	0.0154
Study1	AVS PVS	0.0208	0.0258	3.5106	18	0.0025	0.0083	0.0332
Study2	FPT SMT	-0.0220	0.0241	-3.6632	15	0.0023	-0.0349	-0.0092
Study2	FPT IPT	-0.0093	0.0141	-2.6190	15	0.0194	-0.0168	-0.0017
Study2	FPT AVS	0.0278	0.0274	4.0583	15	0.0010	0.0132	0.0423
Study2	FPT PVS	0.0266	0.0306	3.4723	15	0.0034	0.0103	0.0429
Study2	SMT IPT	-0.0142	0.0156	-3.6398	15	0.0024	-0.0226	-0.0059
Study2	SMT AVS	-0.0270	0.0271	-3.9849	15	0.0012	-0.0414	-0.0126
Study2	SMT PVS	-0.0198	0.0264	-2.9927	15	0.0091	-0.0339	-0.0057
Study2	IPT AVS	-0.0019	0.0144	-0.5134	15	0.6151	-0.0095	0.0058
Study2	IPT PVS	-0.0046	0.0158	-1.1579	15	0.2650	-0.0130	0.0038
Study2	AVS PVS	0.0125	0.0355	1.4118	15	0.1784	-0.0064	0.0315

SUPPLEMENTARY TABLE 16

Paired measure t-test was performed on the difference between Encode and Maintain over the inter-FC clusters.

3.2 ANALYSIS OF INDIVIDUAL DG ROIS

Name	Term	FStat	DF1	DF2	pValue	bonf p
mean Occipital Sup R	load	3.4020	2	54	0.0406	0.5942
mean Parietal Sup L	load	11.6151	2	54	0.0001	0.0017
mean Occipital Mid R	load	2.0607	2	54	0.1373	1.3726
mean Occipital Mid L	load	1.7364	2	54	0.1859	1.6727
mean Supp Motor Area L	load	5.5216	2	54	0.0066	0.1316
mean Occipital Sup L	load	2.9058	2	54	0.0633	0.8229
mean Precentral L	load	0.2365	2	54	0.7902	1.3170
mean Precentral L 1	load	18.7921	2	54	0.0000	0.0000
mean Parietal Sup R	load	10.4143	2	54	0.0001	0.0039
mean Frontal Inf Oper R	load	2.8018	2	54	0.0695	0.8345
mean Fusiform L	load	1.1182	2	54	0.3343	2.0297
mean Fusiform R	load	5.8659	2	54	0.0050	0.1089
mean Lingual R	load	1.0856	2	54	0.3450	2.0060
mean Precentral L 2	load	26.9557	2	54	0.0000	0.0000
mean Frontal Sup 2 R	load	7.4331	2	54	0.0014	0.0338
mean Parietal Inf L	load	14.1915	2	54	0.0000	0.0003
mean Postcentral L	load	0.4926	2	54	0.6138	1.8413
mean Temporal Inf R	load	1.2669	2	54	0.2900	2.2094
mean Lingual L	load	4.9088	2	54	0.0110	0.2089
mean Lingual R 1	load	3.6695	2	54	0.0320	0.5127
mean Postcentral L 1	load	3.4295	2	54	0.0396	0.5942
mean Calcarine R	load	5.6696	2	54	0.0058	0.1222
mean Thalamus L	load	6.2341	2	54	0.0037	0.0843
mean Insula L	load	25.6473	2	54	0.0000	0.0000
mean Insula R	load	14.3226	2	54	0.0000	0.0003
mean Calcarine L	load	2.3735	2	54	0.1028	1.1309
mean Thalamus R	load	4.0788	2	54	0.0224	0.3808
mean Thalamus R 1	load	0.4210	2	54	0.6585	1.8413
mean Thalamus L 1	load	1.3179	2	54	0.2762	2.2094
mean Rolandic Oper L	load	1.0613	2	54	0.3531	1.7249
mean SupraMarginal R	load	27.7152	2	54	0.0000	0.0000
mean Frontal Inf Tri L	load	4.3339	2	54	0.0180	0.3234
mean Putamen L	load	8.6588	2	54	0.0005	0.0137

SUPPLEMENTARY TABLE 17

Maintain specific ROI (using the DG set) activation load effects.

3.3 Load related changes in activity and connectivity as a function of stage

Here we averaged across the three clusters (FPT, AVs and PVS) separately for activity, inter-cluster FC and intra-cluster FC), and then standardized each measure independently. Then we used repeated measures ANOVA where the within factors were activity/connectivity and load. There was no significant main effect for activity/connectivity ($F_{2,36} = 0.0001$, $p = 1$), a strong main effect for load ($F_{2,36} = 10.52$, $p = 0.0011$) and a significant effect for the interaction between load and activity/connectivity ($F_{4,72} = 11.93$, $p < 0.0001$). Further analysis showed that this interaction was driven by a robust effect of load for both connectivity measures ($F^{inter} = 7.05$, $p = 0.0027$; $F^{intra} = 22.56$, $p < 0.0001$;) but not the activity measures. Specifically, inter-FC was significantly greater for medium vs. low load ($t = 3.42$, $p < 0.01$) and there was a threshold-level effect for high vs. medium load ($t = 2.59$, $p = 0.0547$). Similarly, intra-FC was significantly greater for both medium vs. low load ($t = 5.13$, $p < 0.0005$) and high vs medium load ($t = 5.67$, $p < 0.0001$). Similar effects were found in Study 2 for the same analysis. These findings suggest that maintaining higher WM loads increases network functional connectivity substantially but has little impact on overall network activation levels.

3.3.1 Study one

Terms	SumSq	DF	MeanSq	F	pValueGG
(Intercept)	0.0001	1	0.0001	0.0001	1.0000
Error	6.9423	18	0.3857	1.0000	0.5000
(Intercept):metric	0.0001	2	0.0001	0.0001	1.0000
Error(metric)	15.6251	36	0.4341	1.0000	0.5000
(Intercept):load	7.4459	2	3.7230	10.5245	0.0011
Error(load)	12.7347	36	0.3538	1.0000	0.5000
(Intercept):metric:load	5.7688	4	1.4422	11.9262	0.0001
Error(metric:load)	8.7068	72	0.1210	1.0000	0.5000

SUPPLEMENTARY TABLE 18

Repeated measures two-way ANOVA was used to interrogate the effects of load over metric in study one.

Terms	SumSq	DF	MeanSq	F	pValueGG
(Intercept)	0.0001	1	0.0001	0.0001	1.0000
Error	7.0308	18	0.3906	1.0000	0.5000
(Intercept):load	0.2139	2	0.1070	0.7448	0.4366
Error(load)	5.1695	36	0.1436	1.0000	0.5000

SUPPLEMENTARY TABLE 19

Repeated measures one way ANOVA was used to interrogate the effects of load for activation in study one.

Terms	SumSq	DF	MeanSq	F	pValue
(Intercept)	0.0001	1	0.0001	0.0001	1.0000
Error	7.9262	18	0.4404	1.0000	0.5000
(Intercept):load	3.3578	2	1.6789	7.0472	0.0027
Error(load)	8.5764	36	0.2383	1.0000	0.5000

SUPPLEMENTARY TABLE 20

Repeated measures one way ANOVA was used to interrogate the effects of load for inter-connectivity in study one.

load 1	load 2	Difference	StdErr	t	pValue	Lower	Upper
High	Low	0.4892	0.1884	2.5970	0.0547	-0.0079	0.9863
High	Med	-0.0480	0.1229	-0.3914	1.0000	-0.3722	0.2761
Low	High	-0.4891	0.1884	-2.5969	0.0547	-0.9862	0.0080
Low	Med	-0.5372	0.1571	-3.4204	0.0092	-0.9517	-0.1227
Med	High	0.0481	0.1229	0.3915	1.0000	-0.2760	0.3723
Med	Low	0.5373	0.1571	3.4205	0.0092	0.1228	0.9518

SUPPLEMENTARY TABLE 21

Multicomparison post hoc test (Bonferroni corrected) was used to interrogate the significant main effect of load found in the Repeated measures ANOVA for inter-connectivity.

Terms	SumSq	DF	MeanSq	F	pValue
(Intercept)	0.0001	1	0.0001	0.0001	1.0000
Error	7.6105	18	0.4229	1.0000	0.5000
(Intercept):load	9.6431	2	4.8216	22.5553	0.0001
Error(load)	7.6956	36	0.2138	1.0000	0.5000

SUPPLEMENTARY TABLE 22

Repeated measures one way ANOVA was used to interrogate the effects of load for intra-connectivity in study one

load 1	load 2	Difference	StdErr	t	pValue	Lower	Upper
High	Low	0.9528	0.1680	5.6736	0.0001	0.5096	1.3960
High	Med	0.1927	0.1318	1.4621	0.4830	-0.1550	0.5404
Low	High	-0.9527	0.1680	-5.6735	0.0001	-1.3959	-0.5095
Low	Med	-0.7601	0.1482	-5.1309	0.0003	-1.1510	-0.3691
Med	High	-0.1926	0.1318	-1.4620	0.4830	-0.5403	0.1551
Med	Low	0.7602	0.1482	5.1310	0.0003	0.3692	1.1511

SUPPLEMENTARY TABLE 23

Multicomparison post hoc test (bonferroni corrected) was used to interogate the significant main effect of load found in the Repeated measures ANOVA for intra-connectivity.

3.3.2 STUDY TWO

Terms	SumSq	DF	MeanSq	F	pValueGG
(Intercept)	0.0001	1	0.0001	0.0001	1.0000
Error	5.2347	15	0.3490	1.0000	0.5000
(Intercept):metric	0.0001	2	0.0001	0.0001	1.0000
Error(metric)	9.0071	30	0.3003	1.0000	0.5000
(Intercept):load	2.4252	1	2.4252	5.5895	0.0320
Error(load)	6.5083	15	0.4339	1.0000	0.5000
(Intercept):metric:load	1.6405	2	0.8203	3.9232	0.0613
Error(metric:load)	6.2723	30	0.2091	1.0000	0.5000

SUPPLEMENTARY TABLE 24

Repeated measures ANOVA was used to interrogate the effects of load over stage for activation in study two.

load 1	load 2	Difference	StdErr	t	pValue	Lower	Upper
High	Low	0.3179	0.1345	2.3643	0.0320	0.0313	0.6045
Low	High	-0.3178	0.1345	-2.3642	0.0320	-0.6044	-0.0312

SUPPLEMENTARY TABLE 25

Multicomparison post hoc test (Bonferroni corrected) was used to interrogate the significant main effect of load found in the Repeated measures ANOVA.

metric	load 1	load 2	Difference	t	StdErr	pValue	Lower	Upper
BA	High	Low	0.0370	0.4846	0.0763	0.6350	-0.1255	0.1995
BA	Low	High	-0.0369	-0.4845	0.0763	0.6350	-0.1994	0.1256
Inter	High	Low	0.2502	1.2458	0.2009	0.2320	-0.1778	0.6783
Inter	Low	High	-0.2501	-1.2457	0.2009	0.2320	-0.6782	0.1779
Intra	High	Low	0.6666	2.7130	0.2457	0.0161	0.1429	1.1902
Intra	Low	High	-0.6665	-2.7129	0.2457	0.0161	-1.1901	-0.1428

SUPPLEMENTARY TABLE 26

Multicomparison post hoc test (Bonferroni corrected) was used to interrogate the significant interaction of load by stage found in the Repeated measures ANOVA.

4 DECODING WM FACTORS

We used a multivariate classifier over the averaged stacks from both activation and connectivity measures to estimate the decoding performance of processing stages or visual domains using a dense (multi-class SVM) algorithm.

set	factor	metric	GroupCount	Null	oob	CV	Test
DG	Domain	BA	1000	33.3377	85.7968	82.3358	70.5991
DG	Domain	PPI	1000	32.5999	90.3654	86.5505	85.7778
DG	Stage	BA	1000	31.8465	98.6538	96.3266	91.3703
DG	Stage	PPI	1000	33.4111	91.7972	91.9802	87.5856
SG	Domain	BA	1000	32.5326	66.6699	58.1029	75.1937
SG	Domain	PPI	1000	33.0023	82.8266	76.8833	83.6021
SG	Stage	BA	1000	32.3974	93.5795	86.4755	75.8919
SG	Stage	PPI	1000	32.2003	84.4398	75.0221	76.1367
WB	Domain	BA	1000	32.3911	87.6760	79.8539	91.7500
WB	Domain	PPI	1000	31.8702	85.2778	81.5539	87.9257
WB	Stage	BA	1000	31.9712	99.2968	98.4365	90.8931
WB	Stage	PPI	1000	31.5735	92.3563	90.2065	81.1832

SUPPLEMENTARY TABLE 27

Mean performance of different classification models

Metric	Factor	ROI set	mean F1	std F1	MannWhitneyR	p value	CI	
BA	Domain	DG	70.5991	5.9089	1.2239	0.0010	67.9390	73.2593
BA	Domain	SG	75.1937	5.5335	1.2244	0.0010	72.7025	77.6848
BA	Domain	WB	91.7500	2.5812	1.2244	0.0010	90.5880	92.9120
PPI	Domain	DG	85.7778	4.5418	1.2244	0.0010	83.7331	87.8224
PPI	Domain	SG	83.6021	5.0934	1.2244	0.0010	81.3090	85.8951

PPI	Domain	WB	87.9257	3.8227	1.2244	0.0010	86.2047	89.6467
BA	Stage	DG	91.3703	2.0057	1.2245	0.0010	90.4674	92.2733
BA	Stage	SG	75.8919	2.8228	1.2242	0.0010	74.6210	77.1627
BA	Stage	WB	90.8931	2.3542	1.2245	0.0010	89.8333	91.9529
PPI	Stage	DG	87.5856	3.1877	1.2244	0.0010	86.1505	89.0207
PPI	Stage	SG	76.1367	3.5937	1.2244	0.0010	74.5188	77.7545
PPI	Stage	WB	81.1832	2.3183	1.2244	0.0010	80.1395	82.2269

SUPPLEMENTARY TABLE 28

classification effect size compared to the permuted null effect over metrics (activation and connectivity) Factor and set. Empirical p is calculated as the number of null models that are better than the mean F1 of the true model.

Decoding processing stages demonstrate that information from any of the ROI sets and using either connectivity (Supplementary Figure 4 aiii) or activation (Supplementary Figure 4 aii) is sufficient to classify the processing stages with high accuracy compared to the null model (see Supplementary Table 33 for p-values); specifically, for activation (Acc_{WB} = 90.89; Acc_{DG} = 91.37; Acc_{SG} = 75.89) and connectivity (Acc_{WB} = 81.18; Acc_{DG} = 87.6; Acc_{SG} = 76.14).

Source	SS	df	MS	Chi sq	Prob Chi sq	etaSquared
Groups	2587.2667	1	2587.2667	8.4829	0.0036	0.1438
Error	15407.7334	58	265.6506	NaN	NaN	0.0000
Total	17995.0000	59	NaN	NaN	NaN	0.0000

SUPPLEMENTARY TABLE 29

Kruskal Wallis non-parametric (distribution free) test comparing classification accuracy distance from null between activation and connectivity across sets for decoding Stage ($meanRank_{Activity}$=37.07, $meanRank_{Connectivity}$=23.93)

Group1	Group2	N	rankMean1	rankMean2	Z	p value	Effect Size	Set
BA	PPI	10	15.5000	5.5000	3.7419	0.0002	1.1833	DG
BA	PPI	10	10.2000	10.8000	-0.1889	0.8502	-0.0597	SG
BA	PPI	10	15.5000	5.5000	3.7419	0.0002	1.1833	WB

SUPPLEMENTARY TABLE 30

Post-hoc rank-sum Multicomparison across ROI sets

Decoding visual domains, the hierarchical clustering showed very weak dissociations between the WM domains. Therefore, the question arises as to how accurately they could be decoded based on the activity or connectivity from different ROI-sets (WB, DG and SG sets). Unexpectedly, visual domains also had high classification performance when applying a 3-way classifier for activation (Acc_{WB} = 91.75; $F1_{DG}$ = 70.6; $F1_{SG}$ = 75.2) and connectivity (Acc_{WB} = 87.9; $F1_{DG}$ = 85.8; $F1_{SG}$ = 83.6).

Source	SS	df	MS	Chi sq	Prob Chi sq	etaSquared
Groups	1440.6000	1	1440.6000	4.7233	0.0298	0.0801
Error	16554.4000	58	285.4207	NaN	NaN	0.0000
Total	17995.0000	59	NaN	NaN	NaN	0.0000

SUPPLEMENTARY TABLE 31

Kruskal Wallis non-parametric (distribution free) test comparing classification accuracy distance from null between activation and connectivity across sets for decoding Domain ($meanRank_{Activity}$=25.60, $meanRank_{Connectivity}$=35.40)

Group1	Group2	N	rankMean1	rankMean2	Z	p value	Effect Size	Set
BA	PPI	10	5.5000	15.5000	-3.7418	0.0002	-1.1832	DG
BA	PPI	10	5.8000	15.2000	-3.5150	0.0005	-1.1115	SG
BA	PPI	10	15.5000	5.5000	3.7419	0.0002	1.1833	WB

SUPPLEMENTARY TABLE 32

Post-hoc rank-sum Multicomparison across ROI sets

4.1 Increased accuracy of domain decoding at higher WM load

One possibility, is that as WM demands increase, more "domain general" resources are brought on line. Based on this, one would predict that the classification accuracy will decrease as a function of demand as the level of similarity between activation or connectivity patterns increases. Alternatively, it could be the case that as WM demands increase the brain becomes more finely tuned towards a specialised connectivity state. To test between these alternative possibilities, the classifiers were applied to the single trial data (i.e. load x domain x run x repeat = 1026 events for study 1 and load x manipulation x domain x run x repeat = 768 events for study 2) and compared the performance distributions across loads. We start by showing that even within the resolution of a single trial classification accuracy is high.

set	metric	GroupCount	Null	oob	CV	Test
DG	BA	1000	32.4058	72.1050	63.3777	62.4380
DG	PPI	1000	33.1616	76.2719	70.9536	66.1574
SG	BA	1000	32.9017	60.6104	57.3387	66.5490
SG	PPI	1000	32.9434	65.9535	57.7596	65.8313
WB	BA	1000	33.2587	79.8089	69.2018	72.6093
WB	PPI	1000	33.0120	79.3392	73.8061	75.7230

SUPPLEMENTARY TABLE 33

Mean performance of different classification models (metric and set)

Metric	ROI set	mean F1	std F1	MannWhitneyR	p value	CI	
BA	DG	62.4380	2.5280	1.2244	0.0010	61.2999	63.5761
BA	SG	66.5490	3.8565	1.2243	0.0010	64.8128	68.2852
BA	WB	72.6093	2.2833	1.2244	0.0010	71.5814	73.6373
PPI	DG	66.1574	2.5746	1.2244	0.0010	64.9984	67.3165
PPI	SG	65.8313	2.5511	1.2244	0.0010	64.6828	66.9798
PPI	WB	75.7230	1.8086	1.2244	0.0010	74.9087	76.5372

SUPPLEMENTARY TABLE 34

Classification effect size compared to the permuted null effect over metrics (activation and connectivity) and set. Empirical p is calculated as the number of null models that are better than the mean F1score of the true model.

We then show that classification is substantially better for both medium and high compared to low load, regardless of measure (activity or connectivity), or study.

Source	SS	df	MS	Chi sq	Prob Chi sq	etaSquared
Groups	15809.2667	2	7904.6334	23.1638	0.0001	0.2603
Error	44933.2334	87	516.4740	NaN	NaN	0.0000
Total	60742.5000	89	NaN	NaN	NaN	0.0000

SUPPLEMENTARY TABLE 35

Kruskal Wallis non-parametric (distribution free) test comparing classification accuracy distance from null across ROI sets for decoding Activity across loads ($meanRank_{low}$=26.77, $meanRank_{med}$=55.40,$meanRank_{high}$=54.33).

Group1	Group2	lower limit	mean diff	upper limit	p value
low	med	-44.7816	-28.6333	-12.4850	0.0001
low	high	-43.7149	-27.5666	-11.4183	0.0002
med	high	-15.0816	1.0667	17.2150	1.0000

SUPPLEMENTARY TABLE 36

Post-hoc test Bonferroni corrected for multicomparison

Source	SS	df	MS	Chi sq	Prob Chi sq	etaSquared
Groups	28343.4667	2	14171.7334	41.5289	0.0001	0.4666
Error	32399.0334	87	372.4027	NaN	NaN	0.0000
Total	60742.5000	89	NaN	NaN	NaN	0.0000

SUPPLEMENTARY TABLE 37

Kruskal Wallis non-parametric (distribution free) test comparing classification accuracy distance from null across ROI sets for decoding Connectivity across loads (meanRanklow=20.70, meanRankmed=54.57,meanRankhigh=61.23)

Group1	Group2	lower limit	mean diff	upper limit	p value
low	med	-50.0149	-33.8666	-17.7183	0.0001
low	high	-56.6816	-40.5333	-24.3850	0.0001
med	high	-22.8149	-6.6666	9.4817	0.9690

SUPPLEMENTARY TABLE 38

Post-hoc test Bonferroni corrected for multicomparison

Similar inspection was performed for study 2 using Wilcoxon rank sum test replicating study 1 results, showing a small effect for activation ($Z = -2.1363$, $p < 0.05$, $r = -0.39$), and more than double effect for connectivity ($Z = -4.9897$, $p < 0.0001$, $r = -0.9109$).

Group1	Group2	N	rankMean1	rankMean2	Z	p value	Effect Size
Low	High	30	25.6667	35.3334	-2.1363	0.0327	-0.39

SUPPLEMENTARY TABLE 39

Wilcoxon rank sum non-parametric (distribution free) test comparing low and high classification accuracy distance from null for decoding visual domains using Activity ($meanRank_{low}$=25.67, $meanRank_{high}$=35.33)

Group1	Group2	N	rankMean1	rankMean2	Z	p value	Effect Size
Low	High	30	19.2334	41.7667	-4.9897	0.0001	-0.9109

SUPPLEMENTARY TABLE 40

Wilcoxon rank sum non-parametric (distribution free) test comparing low and high classifi- cation accuracy distance from null for decoding visual domains using Connectivity ($meanRank_{low}$=19.23, $meanRank_{high}$=41.77)

4.2 Decoding visual domains across processing stages

A pertinent question is whether the existing domain specific pattern is sustained across processing stages. To test this, we produced a stack of observations containing subject x domain x stages x run events for each study (Study1;19x3x3x3 = 513, Study2;16x2x3x3 = 288) and used the same cross-study approach as above to assess the model's performance. Separating events by stage resulted in a substantial reduction in performance; nonetheless, all models had high accuracy compared to the null (see Supplementary Table 48,49); specifically, for activation ($F1_{WB}$ = 80.9; $F1_{DG}$ = 69.6; $F1_{SG}$ = 74.2) and connectivity ($F1_{WB}$ = 82.46; $F1_{DG}$ = 76.39; $F1_{SG}$ = 74.48). These results suggest that there are persistent regional activity and connectivity patterns that encode for WM domains consistently across the different stages of a trial.

set	metric	GroupCount	Null	oob	CV	Test
DG	BA	1000	30.7761	73.7856	66.7149	69.6238
DG	PPI	1000	33.5074	82.9559	73.9340	76.3889
SG	BA	1000	32.0249	62.4015	56.1749	74.2017
SG	PPI	1000	32.8793	73.5609	64.3746	74.4846
WB	BA	1000	33.4217	82.4025	70.7629	80.8845
WB	PPI	1000	32.5735	84.6021	76.6661	82.4605

SUPPLEMENTARY TABLE 41

Mean performance of different classification models

Metric	ROI set	mean F1	std F1	MannWhitneyR	p value	CI	
BA	DG	69.6238	3.5442	1.2244	0.0010	68.0282	71.2194
BA	SG	74.2017	3.7237	1.2244	0.0010	72.5253	75.8781
BA	WB	80.8845	3.2431	1.2244	0.0010	79.4245	82.3446
PPI	DG	76.3889	2.9659	1.2244	0.0010	75.0536	77.7241
PPI	SG	74.4846	2.8460	1.2244	0.0010	73.2034	75.7659
PPI	WB	82.4605	2.4110	1.2244	0.0010	81.3751	83.5459

SUPPLEMENTARY TABLE 42

classification effect size compared to the permuted null effect over metrics (activation and connectivity) and set. Empirical p is calculated as the number of null models that are better than the mean F1 of the true model.

4.3 STAGE AND DOMAIN SPECIFICITY

Our results suggest the existence of a generalized domain specific states across stages, however, it is plausible to assume that the nature of these states is dynamic and thus is affected by processing stage demands. In other words, can we find evidence supporting secondary domain specific states that are stage specific? To test this, we trained independent domain classification models for each stage from the stack above. We then estimated two measures 1) match distance estimating accuracy improvement for same stage events between global and stage specific models. and 2) mismatch distance estimating accuracy decline for mismatched stage events (see methods for details).

Comparing stage specific models (i.e. trained on a specific stage) performance to global models (i.e. trained using events from all stages), it was apparent that in general the global models outperformed the specific models for both the match and mismatch cases. This wasn't the case for both DG and SG connectivity models where a strong improvement was seen for the specific models. Notably, in all models, as expected Match metric outperformed Mismatch, suggesting the existence of subtle patterns that differentiate between domains and are stage-specific.

Metric	Set	CohenD	P	MeanA	MeanB	Specificty	df	t
BA	WB	-1.04	0.03	78.74	80.69	Match	18.00	-2.33
BA	WB	-3.85	0.00	66.32	80.69	Mismatch	18.00	-8.61
BA	WB	3.32	0.00	78.74	66.32	MatchVsMis	18.00	7.42
BA	DG	-1.71	0.00	65.82	69.05	Match	18.00	-3.82
BA	DG	-5.99	0.00	58.64	69.05	Mismatch	18.00	-13.39
BA	DG	4.30	0.00	65.82	58.64	MatchVsMis	18.00	9.61
BA	SG	-4.52	0.00	65.75	74.08	Match	18.00	-10.10
BA	SG	-6.35	0.00	60.33	74.08	Mismatch	18.00	-14.19
BA	SG	2.35	0.00	65.75	60.33	MatchVsMis	18.00	5.26
PPI	WB	-4.96	0.00	77.15	82.41	Match	18.00	-11.09
PPI	WB	-17.08	0.00	56.35	82.41	Mismatch	18.00	-38.20
PPI	WB	13.65	0.00	77.15	56.35	MatchVsMis	18.00	30.51
PPI	DG	2.74	0.00	79.35	76.08	Match	18.00	6.14
PPI	DG	-14.53	0.00	47.56	76.08	Mismatch	18.00	-32.49
PPI	DG	17.17	0.00	79.35	47.56	MatchVsMis	18.00	38.40
PPI	SG	3.14	0.00	78.16	74.02	Match	18.00	7.02
PPI	SG	-6.54	0.00	63.29	74.02	Mismatch	18.00	-14.61
PPI	SG	8.00	0.00	78.16	63.29	MatchVsMis	18.00	17.88

SUPPLEMENTARY TABLE 43

Comparing Match & Mismatch to each other as well as the global model'S performance using 2-way t-test revealed that across domains both stage specific and stage general patterns code for different aspects of WM domains.

4.4 Decoding Manipulation vs maintain

set	factor	metric	GroupCount	Null	oob	CV
DG	mainpulation	BA	1000	48.0019	37.9816	45.3055
DG	mainpulation	PPI	1000	47.6420	43.4682	49.0877
SG	mainpulation	BA	1000	48.0942	42.4013	49.3940
SG	mainpulation	PPI	1000	47.7101	41.7191	46.0078
WB	mainpulation	BA	1000	48.4354	33.4835	43.4735
WB	mainpulation	PPI	1000	47.1568	37.3194	44.7021

SUPPLEMENTARY TABLE 44

Mean performance of different classification models for the manipulation factor on study 2

Metric	ROI set	mean F1	std F1	MannWhitneyR	p value	CI	
BA	DG	45.3055	7.4396	-0.2250	0.6184	41.9562	48.6547
BA	SG	49.3940	7.9305	0.1079	0.4525	45.8237	52.9642
BA	WB	43.4735	8.4875	-0.3845	0.6943	39.6524	47.2945
PPI	DG	49.0877	7.8940	0.1288	0.4246	45.5339	52.6415
PPI	SG	46.0078	9.4681	-0.1334	0.5784	41.7453	50.2702
PPI	WB	44.7021	7.7989	-0.1944	0.5964	41.1911	48.2131

SUPPLEMENTARY TABLE 45

classification effect size compared to the permuted null effect over metrics (activation and connectivity) Factor and set. Empirical p is calculated as the number of null models that are better than the mean F1 score of the true model.
